# PROTOCOL: Accommodation‐based interventions for individuals experiencing, or at risk of experiencing, homelessness

**DOI:** 10.1002/cl2.1103

**Published:** 2020-09-08

**Authors:** Ciara Keenan, Sarah Miller, Jennifer Hanratty, Therese D. Pigott, Peter Mackie, John Cowman, Christopher Coughlan, Jayne Hamilton, Suzanne Fitzpatrick

**Affiliations:** ^1^ Campbell UK and Ireland, Centre for Evidence and Social Innovation Queen's University Belfast Belfast UK; ^2^ College of Education and Human Development Georgia State University USA; ^3^ School of Geography and Planning Cardiff University UK; ^4^ Department of Social Work Health Service Executive Dublin Ireland; ^5^ Institute for Social Policy, Housing, Environment and Real Estate (I‐SPHERE) Heriott Watt University UK

## 1 BACKGROUND

### 1.1 The problem, condition or issue

Homelessness affects individuals who are experiencing life without safe, adequate or stable housing. Conceived in this way, homeless not only describes those individuals who are visibly homeless and living on the street, but also those precariously housed individuals who; stay in emergency accommodation, sleep in crowded or inadequate housing, and those who are not safe in their living environment. Kuhn and Culhane (1998) further classify individuals experiencing homelessness as those who are chronically homeless, those who are transitionally homeless and those who experience episodic bouts of homelessness.

There are causal relationships between various situational and personal factors which lead to an individual experiencing homelessness (Anderson & Christian, 2003; Morse, 1992). Most researchers do agree that important factors include (but are not limited to); a lack of affordable and adequate housing, poverty caused by unemployment or lack of available resources, absence or reduction of health and social services, breakdowns of personal relationships (Crisis, 2020).

Global data suggests that at least 1.6 billion people lack adequate housing (Habitat for Humanity, 2017). In the European context this figure continues to rise across all European Union member states with the exception of Finland where homelessness has been on the decline since 1987 (FEANTSA 2017; Y‐Foundation, 2017).

Without access to housing, individuals are exposed to disease, poverty, isolation, mental health issues, prejudice and discrimination, and are under constant and significant threat to their personal safety. Therefore, having access to safe, stable and adequate housing is internationally recognised as a basic human right (OHCHR, 2009) and is central to developing a population who are living healthy, safe and happy lives.

Individuals who are currently experiencing poorer physical and mental health are overrepresented in the homeless population (Link, 2014). Additionally, for the large population who are currently living without homes they continue to suffer due to social inequalities which are persistent and enduring and continue to widen over time. These social inequalities coupled with poor health make the pathways out of homelessness especially challenging. Some of these obstacles include; inability to hold steady employment (Rosenberg & Kim, 2018), encountering prejudice and discrimination while trying to access services (Ramsay, Hossain, Moore, Milo, & Brown, 2019), and addiction issues (Tsemberis, 2011).

### 1.2 The intervention

Homelessness is recognised as a multifaceted and complex issue and many accommodation‐based approaches have evolved across the globe to incorporate additional support and services beyond delivery of housing while other interventions deliver only temporary housing which is insufficient to meet peoples basic needs.

Interventions included in this review are those which primarily seek to meet the user's accommodation needs through provision of a short‐term shelter and bed or a long‐term home. These interventions may be provided alongside additional support and services. These interventions will be referred to as accommodation‐based approaches/interventions throughout this protocol. Accommodation‐based approaches with or without additional components is not a new phenomenon and stems from a seemingly accidental combination of global ideas, progression of evidence‐based policy and practice, and establishment of welfare states.

Some of the major accommodation‐based interventions are diverse in their approach which makes classification especially difficult. This coupled with inconsistent descriptions of interventions has rendered current categorisations meaningless.

In this protocol we will describe how the review team created a new and meaningful typology to categorise included interventions, however, initially we will briefly describe some of the familiar interventions found in the evidence base that will fall into the new typology. These interventions have been selected as they are well known to policymakers and are mainly representative of interventions targeting those vulnerable to homelessness, however there is often an inconsistent understanding of what they may look like on the ground in different contexts. For example, although the intervention may be called “Housing First,” there are often discrepancies in how this intervention in implemented across countries and contexts. This section aims to clarify what the main interventions are:

#### 1.2.1 Housing first (HF)

HF interventions offer housing to homeless individuals with minimal obligation or preconditions being placed upon the participant. HF programmes share some common themes: (a) the participant is provided access to permanent housing immediately, without conditions, (b) decisions around the location of the home and the services received are made by the client, (c) support and services to aid the individual recovery are provided alongside housing placement, (d) social integration with local community and meaningful engagement with positive activities is encouraged. HF is based on the principle that housing should be made available in the first instance and preconditions such as sobriety and involvement in treatment programmes are unnecessary barriers placed upon homeless individuals. Through the removal of these common obstacles, it is believed that the individual has a better chance of achieving stabilisation in appropriate housing and feeling more willing or able to accept treatment.

#### 1.2.2 Hostels

Hostels provide accommodation for both short‐term housing needs. Homeless hostels often impose strict rules on the persons who stay there relating to abstinence, behaviour and curfews. The individuals who frequent hostels vary but may include homeless individuals, homeless families, homeless couples and homeless individuals with pets. Sleeping arrangements are variable with some offering dormitory style sleeping alongside communal kitchen, living and shower areas while others have bedsit flats. The type of support offered by a homeless hostel varies, often determined by the resources available and individuals they are able to house. However, some common types of support offered in homeless hostels include a support plan to move to more stable accommodation, practical help with form filling and obtaining necessary governmental documents, or treatment for substance abuse issues.

#### 1.2.3 Shelters

Homeless shelters are a basic form of temporary accommodation where a bed is provided in a shared space overnight. One of the key features of a homeless shelter is that it is transitory and not usually seen as stable forms of accommodation as the individual is often subject to overcrowding, physical altercations, theft, substance abuse, and unhygienic sleeping conditions. Similarly to hostels, homeless shelters often place additional requirements on potential users including night time curfews. Additional services that may or may not be provided by the homeless shelter are warm meals for dinner and breakfast or support from volunteers who help individuals make connections to other services.

#### 1.2.4 Supported housing

Supported housing is an extremely complex intervention type. To be categorised as supported housing, the intervention will combine housing with additional supportive services as an integrated package. The housing offered can be permanent or temporary; nonabstinent contingent or abstinent‐contingent; staffed group homes, community based or in a private unit; and the subsidies towards rent also vary. Supportive services will be offered directly to the individual or through referrals to the relevant body. Supportive services might include those to help with mental health issues, substance misuse, those interventions which increase access to health services, support to continue education or find employment, help with accessing benefits, or those services which focus on social aspects of the individual's life such as positive interactions with society, or community engagement.

Suttor (2016) argues that while it may be advantageous to create interventions tailored to the individual's unique needs, there is a need to classify approaches. Indeed, most commentators acknowledge the challenges of lack of clear definition of the many terminologies used to describe accommodation‐based interventions. One example of this is highlighted in a study which identified 307 unique terms across 400 articles on supported accommodation (Gustafsson et al., 2009 cited in McPherson, Krotofil, & Killaspy, 2018). Additionally, the HF model initially seems like an approach where categorisation is straightforward, however, there exists significant inconsistencies regarding implementation. Various researchers observe that this may be due to the way the HF model has deviated from the original “Pathways to Housing” intervention (Tsemberis & Eisenberg, 2000) due in part to the progression of services and support (Johnson, Parkinson, & Ahuri, 2012; Phillips et al., 2011).

Due to these inconsistencies in the literature it became apparent that the review team must create meaningful categorisations for accommodation‐based interventions to allow functional and useful comparison between various intervention types. The importance of these categorisations cannot be understated, as it provides an international framework from which policy makers and funders can work to provide change on homelessness. Furthermore, it takes an evidence‐based approach to identify what accommodation interventions work best for individuals experiencing homelessness and what components make them most effective.

To develop the typology further, we selected a random sample of five accommodation‐based interventions included in the evidence and gap map (EGM) of homelessness interventions, (White, Saran, Teixeira, Fitzpatrick, & Portes, 2018) upon which this review is based. Second, two review team members then independently coded the characteristics, hypotheses and concepts related to each intervention and compared notes when each reviewer had completed their five papers. This independent analysis of the sampled papers ensured both objectivity and consistency in this step of the process and allowed the reviewers to investigate substantial amounts of data without bias or a predetermined hypothesis. Third, emerging themes were collated, and reviewers communicated to better understand the patterns which appeared through the sampled studies. Finally, through this iterative process we conclude that the most suitable way to create meaningful categorisations would be based around the intensity (defined as the level of the support offered) of the intervention and the expectations posited to the client during it as there was significant diversity in approaches taken.

One such taxonomy already exists and is based on an international evidence review of 533 interventions on rough sleepers. This review was led by one of the current review authors (Mackie & Wood, 2017) and was created to differentiate between types of temporary accommodation, namely shelters and hostels. The review team adapted this taxonomy to help create categorisation for the network of accommodation‐based interventions alongside Lipton and colleagues' (Lipton, Siegel, Hannigan, Samuels, & Baker, 2000) descriptive categorisation of low, moderate, or high intensity housing which is based on the amount of structure and level of independence offered to their 2,937 study participants. A further category (Housing only) was added to fit interventions which focused on giving the individual accommodation for an extended period of time without further support or services offered. It was deemed to be more than just meeting the basic needs of the individual, but not intense enough to meet the criteria of the moderate category, as they were not receiving any additional services or help.

Furthermore, interventions varied on the conditions the client was required to abide by. These conditions include needing to be sober from alcohol and/or drugs, abstain from criminal activity or to gain employment after a certain amount of time. To accurately incorporate these into the categories, it must be stated whether the intervention required such a behavioural condition (conditional) or whether there were no behavioural conditions imposed (unconditional). The typology is as follows:

##### 1.2.4.1 Basic/conditional

Interventions that meet the client's basic human needs only. This would be the provision of a bed and other basic subsistence such as food. There are no named additional services or support offered to the client. This type of intervention focuses more on the short‐term benefit to the client. The accommodation or support offered may require further conditions from the client upon admission such as sobriety or punctuality. An example of this intervention type would be if clients were given one night in a hostel with a meal on the condition that they arrive by 11 pm.

##### 1.2.4.2 Basic/unconditional

Interventions which offer only minimal sleeping facilities to the client without additional services or support. Unlike the type of intervention describe above, there are no behavioural expectations placed on the individual. An example of this would be if clients were provided access to a shelter without exception.

##### 1.2.4.3 Housing only/conditional

The clients are provided a form of discounted or free accommodation for an extended period, with conditions, but without additional support or services. An example of this is shown in Siegel et al. (2006): one of the interventions described provide the participants with housing where they are helped to pay for it financially by their own specific agency. Tenants were responsible for their own meals and utility expenses. An example of the behavioural expectations imposed on clients receiving this type of intervention may be that they must enter paid employment within 6 months.

##### 1.2.4.4 Housing only/unconditional

Provision of housing for an extended period but without further support and services offered to the client. The participant is not required or obligated to meet any behavioural expectation to retain their housing.

##### 1.2.4.5 Moderate support/conditional

Moderate levels of support and/or services are provided in addition to housing. The level of support and type of service offered will remain general and aimed towards the homeless population as a single entity, and not specific to individual personal needs. This housing coupled with general support and services will be offered on the condition that an individual meets a behavioural expectation. For example, in Sosin, Bruni and Reidy (1996), a housing intervention alongside a moderately intensive drug case management intervention was offered. To take part, participants had to sign a contract agreeing to abstain from drugs and or alcohol.

##### 1.2.4.6 Moderate support/unconditional

Interventions in this category are the same as the above category except there will not be a behavioural expectation placed on the client. For example, Lim et al. (2017) focused on accessing cheaper housing and services to prevent youth from becoming homeless. The participants were encouraged to attend but it was not strictly enforced and there were no conditions placed upon the individuals to partake in the intervention.

##### 1.2.4.7 High support/conditional

These interventions provide housing and actively and assertively work to improve client's long‐term outcomes. The intervention provides assertive, individualised services and interventions for clients. They often focus specifically on the personal needs of the client. The intervention can involve improving housing stability, health, and employment, among other specific needs. The accommodation or support offered may place a behavioural expectation upon the person upon admission to the intervention. For example, participants in Schumacher et al. (2003) were provided housing alongside intensive treatment and other services. All participants were routinely tested for drugs and alcohol and were not allowed to continue with the intervention until were they deemed sober.

##### 1.2.4.8 High support/unconditional

Interventions in this category are the same as the above category except there will not be a behavioural expectation placed on the client. For example, Levitt et al. (2013) intervention included providing housing, meals and on‐site care services. On‐site case managers would consistently work with each individual participant on their substance use and life goals. The participant did not need to be sober to partake in the intervention.

##### 1.2.4.9 No intervention

Interventions in this category would be those that do not actively work to improve the lives of the clients. The client is not offered a bed/food or any additional support by the researchers. An example of this is shown in Sosin et al. (1996) article. The control group used in this experiment received no additional aid from those conducting it. Those in the control received some minimal information on where they could receive help in the form of abuse agencies or welfare offices but were not offered any additional help or services by researchers.

### 1.3 How the intervention might work

The distinctive component shared by all accommodation‐based interventions is that accommodation will be provided to individuals (even if only for the short‐term). Some interventions may also provide accommodation alongside the service and support they require to continue life independently without the risk of future homelessness. By providing accommodation, individuals will have a greater opportunity to concentrate their efforts on gaining support to address other areas of their lives, for example, in health care, education or employment. As suggested in the new typology, accommodation programmes may provide additional supportive services, creating more opportunities for individuals to access services onsite where they live. This integrated support can importantly provide necessary individualised services within a familiar and welcoming context. The intensity of the intervention is also related to this; if the intervention provides intensive individualised support, the individual is more likely to engage and take advantage of the services available.

Regarding conditionality, if certain conditions such as sobriety or compulsory attendance are required as part of the accommodation agreement, this can also increase engagement with services or improve the individuals health outcomes. However, conditionality can be detrimental to individualised entrenched in homelessness, as they may be unwilling to change their situation without ownership over the decision.

### 1.4 Why it is important to do this review

The aim of this systematic review and network meta‐analysis is to establish the effectiveness of accommodation‐based approaches though a robust and rigorous synthesis of the available literature.

The network meta‐analysis will also allow us to rank the effectiveness of interventions according to the categorisations described in the typology outlined earlier. Study characteristics will be examined through moderator analysis and investigation of potential heterogeneity. Through investigation of the sources of variance, review authors can explain potential differences in effect sizes. This will be particularly important in the field of homelessness research which embraces a complex systems perspectives and experts are not only drawn to a “what works” linear cause and effect but also towards an understanding of what works, for whom, and in what circumstances?

#### 1.4.1 Previous reviews

This systematic review will be based on evidence already identified in two existing EGMs commissioned by the Centre for Homelessness Impact (CHI) and built by White et al. (2018). The EGMs present studies on the effectiveness and implementation of interventions aimed at people experiencing, or at risk of experiencing, homelessness.

The EGMs identified various systematic reviews which assess the effectiveness of interventions like HF (Beaudoin, 2016; Woodhall‐Melnik & Dunn James, 2016) and supported housing (Burgoyne, 2013; Nelson, Aubry, & Lafrance, 2007; Richter & Hoffmann, 2017), and interventions which were conducted in hostel and shelter settings (Haskett, Loehman, & Burkhart, 2016; Hudson, Flemming, Shulman, & Candy, 2016). However, a network meta‐analysis of accommodation‐based interventions for a homeless population does not exist. Various systematic reviews which synthesise accommodation‐based interventions more generally, differ from the proposed review in several ways:

##### 1.4.1.1 Differences in population

Bassuk et al. (2014) systematically reviewed and narratively reported the findings of six studies which looked at the effectiveness of housing interventions and housing combined with additional services. The interventions included HF, rapid rehousing, vouchers, subsidies, emergency shelter, transitional housing and permanent supportive housing. However, authors limited the population to American families who were experiencing homelessness and so any final conclusions on the efficacy of accommodation‐based interventions on the wider population of individuals experiencing homelessness are impossible to reach.

##### 1.4.1.2 Differences in outcomes of interest

Fitzpatrick‐Lewis et al. (2011) conducted a rapid systematic review on the effectiveness of interventions to improve the health and housing status of individuals experiencing homeless which located 84 relevant studies. Only those studies published between January 2004 and December 2009 were included in this review and so the current review will be more current and much broader in scope. Additionally, the primary purpose of the review was to identify literature which improved health outcomes for those experiencing homelessness and so other important outcomes were not included.

A title registration form has been submitted to the Campbell Collaboration by Mathew et al. (2018) which looks at how various interventions impact the physical and mental health of homeless individuals alongside other social outcomes. One objective listed in the title registration form is similar to the scope of the current review. Authors will assess “What are the effects of housing models (i.e. Housing First) on the health outcomes of homeless and vulnerably housed adults compared to usual or no housing?” However, the current review will have a wider scope by including additional outcomes across a wider population.

A recent Campbell Collaboration review by Munthe‐Kaas 2018 assessed the effectiveness of both housing and case management programmes for people experiencing, or at risk of experiencing homelessness. The main outcomes of interest to the authors were reduction in homelessness and housing stability. Authors searched the literature until January 2016 and uncovered 43 randomised controlled trials (RCTs) meeting the predetermined inclusion criteria. Authors did not include qualitative research or extract data related to the cost of the interventions, which are variables of interest to this proposed review.

##### 1.4.1.3 Differences in analytic methods

Finally, a recent review by the what works centre for wellbeing (Chambers et al., 2018) included 90 studies which included clusters of HF (*n* = 47), supported housing (*n* = 12), recovery housing (*n* = 10), housing interventions for ex‐prisoners (*n* = 7), housing interventions for vulnerable youth (*n* = 3) and “other” complex interventions targeted at those with poor mental health (*n* = 11). Authors presented a comprehensive search strategy of both commercial and grey literature, however, due to resource constraints were unable to conduct independent screening of the potential studies and therefore risk selection bias in the review. Additionally, only studies published after 2005 were included in this review and so the current review will be broader in scope. Finally, the authors objective was to create a conceptual pathway and evidence map between housing and wellbeing and so the results were not meta‐analysed but described narratively instead.

Policy makers and practitioners have had a legal and moral responsibility to protect individuals experiencing or at risk of experiencing homelessness from the debilitating effects of living without a home. Due to these responsibilities, many researchers have now attempted to understand which accommodation‐based interventions may work best, for whom, and in which circumstances. Through synthesis of the available and most robust research, this review will provide the best estimation of reality, by combining more data than a primary research study feasibly could.

## 2 OBJECTIVES

1. What is the effect of accommodation‐based interventions on outcomes for individuals experiencing or at risk of experiencing homelessness?
2. Which category of intervention is most/least effective compared to other interventions and compared to business as usual (passive control)?
3. Who do accommodation‐based interventions work best for?
  a) Young people or older adults?
  b) Individuals with high or low complex needs?
  c) Families or single individuals?
4. Does the geographical spread of housing (scattered site or conglomerate/congregate) affect the outcomes experienced by individuals experiencing or at risk of experiencing homelessness?
5. What implementation and process factors impact intervention delivery?

## 3 METHODS

### 3.1 Criteria for considering studies for this review

#### 3.1.1 Types of studies

We will include all study designs where a comparison group was used. This includes RCTs, quasiexperimental designs, matched comparisons and other study designs that attempt to isolate the impact of the intervention on homelessness using appropriate statistical modelling techniques.

As RCTs are accepted as more rigorous than nonrandomised studies, the potential impact of a nonrandomised study design on effect sizes will be explored as part of the analysis of heterogeneity.

Studies are eligible for inclusion in the review if they include an inactive comparison condition, for example:

- No treatment.
- Treatment as usual where people receive their normal level of support or intervention.
- Waiting list where individuals or groups are randomly assigned to receive the intervention at a later date.
- Attention control, where participants receive some contact from researchers but both participants and researchers are aware that this is not an active intervention.
- Placebo where participants perceive that they are receiving an active intervention, but the researchers regard the treatment as inactive.

Studies with no control or comparison group, unmatched controls or national comparisons with no attempt to control for relevant covariates will not be included. Case studies, opinion pieces or editorials will also be excluded.

#### 3.1.2 Types of participants

Homelessness affects individuals who are experiencing life without safe, adequate, or stable housing. Conceived in this way, homelessness not only describes those individuals who are visibly homeless and living on the street, but also those precariously housed individuals who; stay in emergency accommodation, sleep in crowded or inadequate housing and those who are not safe in their living environment. FEANTSA further classify individuals experiencing homelessness as those who are roofless, those who are houseless and those who experience insecure or inadequate housing (Feantsa, 2005).

This systematic review will focus on all individuals who are currently experiencing, or at risk of experiencing homelessness irrespective of age or gender. The included studies will include populations from high‐income countries, as defined by the EGM. Homelessness is defined as those individuals who are sleeping “rough” (sometimes defined as street homeless), those in temporary accommodation (such as shelters and hostels), those in insecure accommodation (such as those facing eviction or in abusive or unsafe environments) and those in inadequate accommodation (environments which are unhygienic and/or overcrowded).

#### 3.1.3 Types of interventions

Interventions will include those based on the typology presented in Table 1. This typology is broad enough to include all accommodation‐based approaches which meet our eligibility criteria. These classifications are based on the nature and characteristics of the intervention and not on the descriptor attached by the study author. Interventions will be tested against either a control group or through head to head comparisons with an alternative treatment. Control groups can include various types, such as; placebo, no treatment, waitlist or usual treatment (standard care).

**Table 1:** Typology summary of categories

	Type of accommodation	Support	Conditionality
Basic/conditional	Interventions that meet the client's basic human needs only, for example, providing bed and other basic subsistence such as food	There are no named additional services or support offered to the client. This type of intervention focuses more on the short‐term benefit to the client	Conditions such as sobriety or punctuality apply
Basic/unconditional	Interventions that meet the client's basic human needs only, for example, providing bed and other basic subsistence such as food	There are no named additional services or support offered to the client. This type of intervention focuses more on the short‐term benefit to the client	Accommodation is not conditional on adherence to rules such as sobriety or punctuality
Housing only/conditional	Discounted or free accommodation for an extended period	Without additional support or services	Behavioural expectations are imposed on clients, for example, they must enter paid employment within 6 months
Housing only/unconditional	Discounted or free accommodation for an extended period	Without additional support or services	The participant is not required or obligated to meet any behavioural expectation to retain their housing
Moderate support/conditional	Discounted or free accommodation for an extended period	The level of support and type of service offered will remain general and aimed towards the homeless population as a single entity, and not specific to individual personal needs	Expectations on behaviour in place for example signing a contract agreeing to abstain from drugs and or alcohol
Moderate support/unconditional	Discounted or free accommodation for an extended period	The level of support and type of service offered will remain general and aimed towards the homeless population as a single entity, and not specific to individual personal needs	Accommodation not conditional on engagement (though engagement may be encouraged)
High support/conditional	Discounted or free accommodation for an extended period	Assertive, individualised services and interventions for clients. They often focus specifically on the personal needs of the client	Expectations such as abstinence from alcohol and drugs in place
High support/unconditional	Discounted or free accommodation for an extended period	Assertive, individualised services and interventions for clients. They often focus specifically on the personal needs of the client	No behavioural expectation such as sobriety placed on the client

#### 3.1.4 Types of outcome measures

This review primarily addresses how interventions can reduce homelessness and increase housing stability for those individuals experiencing, or at risk of experiencing, homelessness.

##### 3.1.4.1 Primary outcomes

Housing stability might be described as: time spent homeless, number of participants housed or time spent in specific residential setting.

##### 3.1.4.2 Secondary outcomes

Secondary outcomes include:

- Access to mainstream healthcare
- Crime and justice
- Employment and income
- Capabilities and wellbeing

These outcomes reflect the domains used in the EGM (White et al., 2018).

##### 3.1.4.3 Types of settings

Settings where these accommodation‐based interventions take place may be varied and might include hostels, shelters, and community housing.

### 3.2 Search methods for identification of studies

This systematic review will be based on evidence already identified in two existing EGMs commissioned by the CHI and built by White et al. (2018). The EGMs present studies on the effectiveness and implementation of interventions aimed at people experiencing, or at risk of experiencing, homelessness in high income countries.

#### 3.2.1 Electronic searches

The maps used a comprehensive three stage search and mapping process. Stage one was to map the included studies in an existing Campbell review on homelessness (Munthe‐Kaas, Berg, & Blaasvær, 2018), stage two was a comprehensive search of 17 academic databases, three EGM databases and eight systematic review databases for primary studies and systematic reviews.

#### 3.2.2 Searching other resources

Finally stage three included web searches for grey literature, scanning reference lists of included studies and consultation with experts to identify additional literature. Sample search terms can be found in the protocol (White et al., 2018).

### 3.3 Data collection and analysis

#### 3.3.1 Description of methods used in primary research

Interventions will include randomised and quasirandomised trials measuring effectiveness of accommodation‐based approaches against either a control group or through head to head comparisons with an alternative treatment.

#### 3.3.2 Criteria for determination of independent findings

Often, authors will report data on the same participants across more than one outcome, this leads to multiple dependent effect sizes within each single study. The meta‐analysis will use robust variance estimation to adjust for effect size dependency. (Hedges, Tipton, & Johnson, 2010). The correction for small samples (Tipton & Pustejovsky, 2015) will be implemented when necessary. Finally, in cases where study authors separate participants into subgroups relating to age, comorbid diagnosis, or gender and its inappropriate to pool their data, these participants will remain independent of each other and will be treated as separate studies which each provide unique information.

#### 3.3.3 Selection of studies

As the inclusion/exclusion criteria for this review will be narrower in scope that the scope of the EGM, the review team will independently screen all studies included in the map to meet the predetermined eligibility criteria outlined previously.

We will not undertake any additional searching. However, if in the course of contacting authors for additional information or data necessary for conducting analysis and risk or bias assessments, authors provide us with additional eligible studies these would be included.

#### 3.3.4 Data extraction and management

##### 3.3.4.1 Details of study coding categories

The studies contained within the exiting EGMs will be screened against the inclusion criteria for eligibility by two independent screeners. Once eligible studies have been found, we will undertake dual data extraction, where two authors will both complete data extraction and risk of bias (ROB) assessments independently for each study. Coding will be carried out by trained researchers. Any discrepancies in screening or coding will be discussed with senior authors until a consensus is reached.

Data extraction sheets have been designed by the authors and piloted by trained research assistants using Eppi‐Reviewer. A copy of the data extraction book is attached in Appendix 1. At a minimum we will extract the following data: publication details, intervention details including setting, dosage and implementation, delivery personnel, descriptions of the outcomes of interest including instruments used to measure, design and type of trial, sample size of treatment and control groups, Data required to calculate Hedge's *g* effect sizes, quality assessment. We will also extract more detailed information on the interventions such as: duration and intensity of the programme, timing of delivery, key programme components (as described by study authors), theory of change.

#### 3.3.5 Assessment of ROB in included studies

Assessment of methodological quality and potential for bias will be conducted using the second version of the Cochrane Risk of Bias tool for RCTs (Higgins et al., 2019). Nonrandomised studies will be coded using the ROBINS‐I tool (Sterne and Egger, 2005).

#### 3.3.6 Measures of treatment effect

##### 3.3.6.1 Statistical procedures and conventions

All analyses will be conducted using the R program. The outcomes related to homelessness are continuous and so the effect size metric chosen is Hedges' *g* which will be calculated from means and standard deviations in the first instance, however, if a study does not provide this raw data, authors will be contacted, and this information will be requested. Failing this, many papers have been published to assist authors in calculating Hedge's *g* from primary research (Rosnow & Rosenthal, 1996; Rosnow, Rosenthal, & Rubin, 2000), and have enabled authors to transform many statistical tests of significance such as *t* tests, *F* tests and *χ* ^2^ values to a metric which allows comprehension of the magnitude of the intervention effect. A very useful online calculator has also been developed, this allows authors to choose the type of raw data available and the calculator will automatically transform this to various effect size types, including Hedge's *g* (Lipsey & Wilson, 2001).

Given the expected variation across studies, we will use the random effects model. We will report the estimate of *τ* ^2^ and the prediction interval for the overall mean effect size. We will use restricted maximum likelihood methods to estimate *τ* ^2^ using the R program *metafor*.

###### 3.3.6.1.1 Network meta‐analysis

A traditional pairwise meta‐analysis allows a researcher to compare the evidence base of intervention A against the evidence base for intervention B to inform decisions on whether intervention A or B (or no treatment if compared to a control condition) is most effective for the population, condition or setting of interest. These meta‐analyses provide direct comparisons between two different interventions.

When two or more intervention types exist, as in the case of accommodation‐based approaches, researchers can utilise all the available direct comparisons between intervention options and use this data to calculate indirect comparisons (see example below). This not only allows researchers to assess whether the combination of multiple accommodation‐based approaches is more effective than using one single approach, but also by this combination of both direct and indirect comparison data, researchers are providing a much stronger and more robust evidence‐base to decision makers.

To answer the research question outlined above, network meta‐analysis (NMA) allows analysis of data collected at various time points that compare accommodation‐based approaches against either a control group or through head to head comparisons.

To illustrate how NMA helps to answer the question on effectiveness of accommodation‐based interventions to reduce homelessness, we will use six fictional randomised control trials uncovered through a thorough systematic review of the literature.

1. Study 1 compares basic conditional (labelled BC) to a control group (labelled CG)
2. Study 2 compares housing unconditional (labelled HU) to a control group
3. Study 3 compares moderate conditional (labelled MC) to control group
4. Study 4 compares basic conditional to high unconditional
5. Study 5 compares moderate unconditional (labelled MU) to moderate conditional
6. Study 6 compares high conditional (labelled HC) to basic conditional

**Figure 1:**
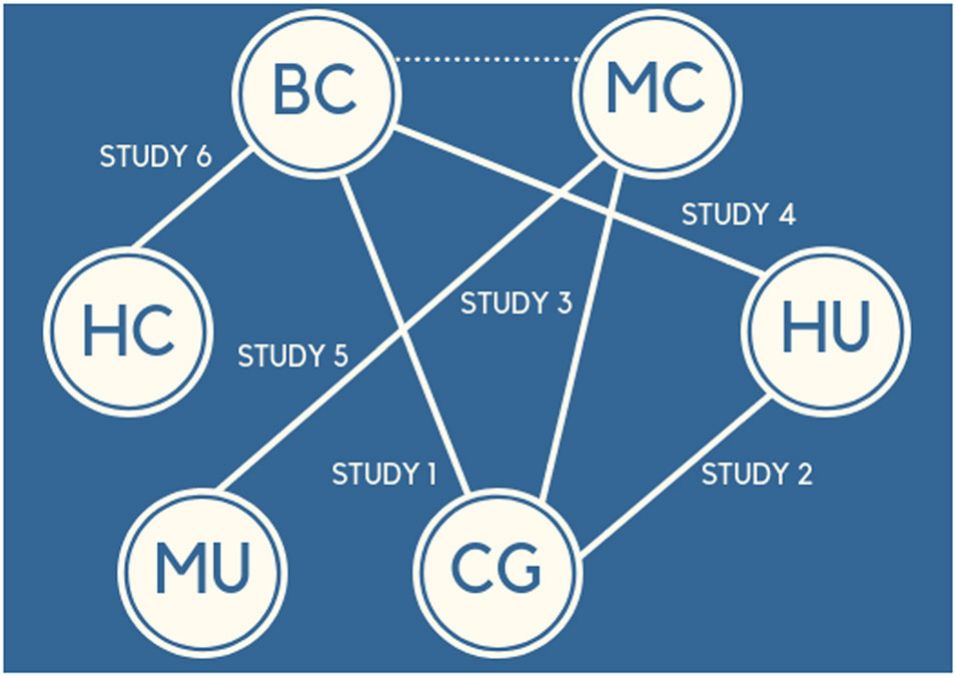
Example network meta‐analysis

The example network meta‐analysis (NMA) would look like this: Figure 1

The NMA can use all the information available across the six studies to provide an understanding of the effectiveness of the approaches. Each line in the diagram is a direct comparison between two interventions and so effect sizes will be available.

However, as shown in the example above, the dashed line between basic conditional and moderate conditional is illustrative of how an indirect comparison (effect size) could be calculated using the information from Study 1 (basic conditional to a control group) and Study 3 (moderate conditional to control group). This indirect effect comparing basic conditional and moderate conditional housing approaches can be calculated because the two interventions of interest have a common comparator (in this case control). If, when the review is updated, a new RCT that compares basic conditional and moderate conditional is located, then this direct effect will be pooled with the earlier indirect effect to create what becomes known as a network treatment effect.

To conclude, the six fictional trials alongside the indirect comparison now create the network of evidence on accommodation‐based approaches. These approaches can now be ranked to provide robust conclusions on which approaches (or combinations of approaches) work best to reduce homelessness.

#### 3.3.7 Unit of analysis issues

We will conduct a network meta‐analysis if there are sufficient studies that meet the transitivity assumption necessary for a network meta‐analysis. Transitivity requires all interventions included in a network meta‐analysis to be jointly randomizable (Salanti, 2012). In other words, transitivity means that the interventions included in the network meta‐analysis could be included in a single randomised, multiarm study. The assumption of transitivity also implies that the any effect size modifiers, characteristics of the studies that may relate to variation across effect sizes, are equivalent across studies. We will use the results of the meta‐regression to examine the heterogeneity across studies, and the balance of potential effect modifiers across studies. If we are able to identify a set of interventions that meet the transtivity requirement, we will conduct a network meta‐analysis to examine comparative effectiveness of those homelessness interventions. The R program *netmeta* will be used for the analysis.

#### 3.3.8 Dealing with missing data

If study reports do not contain sufficient data to allow calculation of effect size estimates, authors will be contacted to obtain necessary summary data, such as means and standard deviations or standard errors. If no information is forthcoming, the study will not be included in meta‐analysis and will be instead included in a narrative synthesis.

#### 3.3.9 Assessment of heterogeneity

The meta‐analysis will include reporting the overall mean and prediction interval for all primary outcomes in the analysis to examine the distribution of effect sizes. The analysis will be conducted in two phases: (a) the use of meta‐regression to examine heterogeneity across studies, and (b) if possible, a network meta‐analysis to address the relative effects of the included interventions.

#### 3.3.10 Assessment of reporting biases

A funnel plot and Egger's linear regression test will be included to check for publication bias across included studies (Sterne & Egger, 2005). Where the funnel plot is asymmetrical this indicates either publication bias or bias which relates to smaller studies showing larger treatment effects. The trim and fill method will be used where the funnel plot is asymmetrical (Higgins et al., 2019), this is a nonparametric technique which removes the smaller studies causing irregularity until there is a new symmetrical pooled estimate, the studies which were eliminated where then filled back in to reflect the new estimate.

#### 3.3.11 Data synthesis

Briefly describe the statistical analysis plan for the review.

#### 3.3.12 Subgroup analysis and investigation of heterogeneity

We will conduct moderator analyses on outcomes to examine the variation across studies in the effectiveness of accommodation‐based interventions. We will use the R programmes metafor (Viechtbauer, 2010) for analyses, netmeta for NMA (Rücker, Schwarzer, Krahn, & König, 2015), and clubSandwich (Pustejovsky, 2017) to adjust the standard errors of the model for dependencies. The intended moderators for subgroup analyses include: participant age, complexity of need, whether the intervention was focused on families or individuals, geographical spread of housing (scattered site or conglomerate), study design, and ROB.

To ensure robustness of the review and to account for individual studies that appear to exert an undue influence on findings, process sensitivity analysis will also be carried out on domains relating to the quality of the included studies.

#### 3.3.13 Sensitivity analysis

##### 3.3.13.1 Treatment of qualitative research

The qualitative research included in this review is based upon existing evidence collated through an EGM constructed by White et al. (2018) and White, Wood, & Fitzpatrick (2018). The EGM was commissioned by the CHI and presents 292 qualitative process evaluations on the implementation issues of interventions designed to target homelessness. These were screened on May 10th, 2019 for duplicates.

The categories included in the EGM describe the factors that impact upon interventions and the implementation of these across the gathered studies. These categories were developed using an iterative process and were initially based on the implementation science framework (Aarons, Hurlburt, & Horwitz, 2011). The categories were then independently piloted against process evaluations and agreement was reached by researchers in the Campbell Collaboration, Campbell UK and Ireland, and Herriot‐Watt University. The five broad categories agreed are contextual factors, policy makers/funders, programme managers/implementing agency, staff/case workers, and recipients. The review team recognise that in the majority of interventions, more than one of the agreed categories could act as a factor that impacts positively or negatively on the effectiveness of the intervention, or both in some cases. This potential overlap reflects the complexity of the implementation of the interventions and the multifaceted evaluation tools needed within this review. For this reason, the review team have decided to focus on one domain in order to formulate a coherent thematic synthesis of the available qualitative data.

In the relevant interventions available for meta‐analysis, process evaluations of these interventions have been identified by the EGM, some of which will be included in a thematic synthesis of qualitative data. A process evaluation aims to examine how well the programme is working and if its implementation followed the intended design. Qualitative evidence that examines the detail of how an intervention is delivered, accessed and experienced by providers and service users enable us to answer questions about why an intervention works (or does not work), who it works for and under what circumstances. This can be used to inform programme and intervention development and service improvement.

We will include process evaluations and other relevant qualitative studies that provide data that enables a deeper understanding of why an intervention does (or does not) work as intended, for whom and under what circumstances. We will conduct a thematic synthesis, as explained by Thomas and Harden (2008), describing the characteristics of included qualitative studies in terms of what qualitative methods have been used to capture this rich data, the number of interviews/focus groups/observations that have taken place, who participated and the nature of qualitative data collection (type and time taken). For example, Tinland et al. (2013) make direct observations on participants but additionally carry out in depth interviews and focus groups with policy makers and practitioners. Similarly, Luffborough (2017) carried out a mixed methods study by administering pre and posttest surveys to 108 homeless men, observing their participation in programme activities and interviewing a sample of 10 on their perceptions of the intervention. The implementation and process evaluations will be critical in this analysis, and data gathered from observations, focus groups and interviews will add an essential and unique human perspective to this review. By including an element of qualitative evidence synthesis in our review we hope to provide a more robust and rich review of the evidence base.

The quality of these mixed methods studies will be assessed using a tool developed by White and Keenan (2018). Along with the tool, the review team intend to use a thematic synthesis methodology to generate new themes and create meaningful relationships between these themes (Fleming, Booth, Garside, Noyes, & Tunçalp, 2019). The tool is similar to the fidelity assessment used by Stergiopoulos and Politis (2013) and aims to provide an accurate account of the eligible qualitative studies. The tool will consider methodology, recruitment and sampling, bias, ethics, analysis and findings, therefore providing a compelling justification for the inclusion of qualitative data. This tool will capture the factors that impact upon intervention effectiveness which can be viewed through the lens of all perspectives. For example, within the context of service delivery politics, policies, welfare and healthcare systems. Similarly, fidelity and implementation problems can impact upon the effectiveness of the intervention. From the perspective of the service user, who can access the services along with the barriers and facilitators of uptake will also impact on the effectiveness of the intervention. The experience that the service user receives in terms of acceptability and dropout rate will cause additional impact. All of these factors of impact along with lessons learnt by Soilemezi and Linceviciute (2018) will be carefully considered during the process of thematic synthesis.

## CONTRIBUTIONS OF AUTHORS

The review will be undertaken by systematic review specialists within the Campbell UK and Ireland Centre. S. M. will be the principal investigator of the project and will have overall responsibility for its conduct and delivery. C. K. will be responsible for the day to day operation of the review. This review will be supported by specialist input from J. H., Terri Pigott, P. M., J. C., and S. F. alongside research support from two full time research assistants, C. C. and J. H.

- Content: C. K., P. M., C. C., J. H., J. C., and S. F.
- Systematic review methods: C. K., S. M., J. H., and T. P.
- Statistical analysis: C. K., S. M., J. H., T. P.
- Information retrieval: EGM by White et al. (2018)

## DECLARATIONS OF INTEREST

The authors declare that there are no conflict of interests.

## PRELIMINARY TIMEFRAME

September 2020

## PLANS FOR UPDATING THIS REVIEW

Dependant on additional funding

## APPENDIX A DATA COLLECTION FORM FOR HOMELESSNESS REVIEWS

**Table:**

1. Bibliographic information	
Article ID	FREETEXT
Linked articles	FREETEXT
Extracted by	FREETEXT
Checked by	FREETEXT
Year of publication	FREETEXT
Type of publication	1. Journal article 2. Book/book chapter 3. Government report 4. Conference proceedings 5. Presentation 6. Thesis or dissertation 7. Unpublished report 8. Other (please specify)
Location of study	1. UK 2. ROI 3. Rest of Europe 4. United States 5. Canada 6. South America 7. Central America 8. Oceania 9. Middle‐East 10. Asia 11. Africa 12. Other (please specify)
*The location in which the study is set **not** where the study authors are based*.	
	Not specified
Study funding sources	1. Research council funding 2. University scholarships and bursaries 3. Salaried research assistantships from university departments 4. Grants or loans from trusts and charities 5. Local enterprise initiatives 6. Company sponsorship 7. Government loans 8. EU Scholarships 9. Industry sponsorship 10. Other (please specify)
Possible conflicts of interest	1. Yes, possible/definite conflict of interest 2. No, study appears to be free of CoI 3. Cannot tell

2. Participant information	
Recruitment setting	1. Clinical setting 2. Accommodation for individuals experiencing homelessness 3. Family home 4. The street 5. Community setting 6. Referred by friends or family 7. Referred by medical health professional 8. Housing agency 9. Other (please specify)
*Where were participants recruited from?*	
Homelessness Status at intake	1. Sleeping “Rough” (or rooflessness) 2. Temporary accommodation 3. Insecure accommodation 4. Inadequate accommodation 5. Involuntary sharing, for example, domestic violence 6. Hidden/concealed homelessness 7. Other (please specify)
*Describe the housing status of the sample at intake and/or any information given about housing status prior to intake. Tick all that apply and try to extract numbers were available*.	
*Homelessness is defined as those individuals who are sleeping* “*rough*” *(sometimes defined as street homeless), those in temporary accommodation (such as shelters and hostels), those in insecure accommodation (such as those facing eviction or in abusive or unsafe environments), and those in inadequate accommodation (environments which are unhygienic and/or overcrowded)*.	
	Not Specified
Geographical context	1. Urban 2. Rural 3. Suburban 4. Mixed 5. Other (please specify)
*Where participants receive treatment?*	
	Not Specified
Gender	FREETEXT
*% (actual number)*	
Age	1. Under 25 2. 25 and over
*Extract mean age, SD and range*.	
*Choose multiple options if the analysis is reported separately for different age groups*.	
Complexity of needs	1. Poor physical health 2. Poor mental health 3. Incarceration 4. Substance abuse issues 5. Care leaver 6. Limited access to integrated support services 7. High risk of harm and/or exploitation 8. Other (please specify)
*What other challenges does the individual face, if any, aside from the risk or experience of homelessness?*	
*High Risk of Harm and/or Exploitation—For example, women in shelters, newcomer families, refugee/asylum seeker, care leavers*	
	Not Relevant
	Not Specified
Mental health status	1. Receiving treatment 2. Not receiving treatment 3. Other (please specify)
	Not relevant
	Not Specified
Substance use status	1. Receiving treatment 2. Not receiving treatment 3. Other (please specify)
	Not relevant
	Not Specified
Homelessness status	1. Sleeping “rough” 2. Temporary accommodation 3. Insecure accommodation 4. Inadequate accommodation 5. Other (please specify)
*Homelessness is defined as those individuals who are sleeping “rough” (sometimes defined as street homeless), those in temporary accommodation (such as shelters and hostels), those in insecure accommodation (such as those facing eviction or in abusive or unsafe environments), and those in inadequate accommodation (environments which are unhygienic and/or overcrowded)*.	
	Not Specified
Family vs. no family	1. Family 2. Nonfamily
*Family = any child involved*	
*Nonfamily = single person or couple without children*	Not Specified
*If mixed sample select both and describe*	
Sample size of treatment group	FREETEXT
*Number of people assigned to treatment. If more than one treatment group extract all and be clear which group is which*.	
Sample size of control group	FREETEXT
*Number of people assigned to control. If more than one control group extract all and be clear which group is which*.	

3. Intervention information	
How many intervention arms in this trial?	FREETEXT
*List how many study arms there are and given each a name. For example, intervention = critical time intervention; control = treatment as usual*	
*If there is more than one intervention arm go to the* “*Study Arm*” *tab and add the RELEVANT study arms. You must then extract data for each relevant study arm*.	
Name of intervention	FREETEXT
*Write in the name of the program, intervention or treatment under study. This may be specific like “critical time intervention” or it may be something more generic like “supported housing*”	
Briefly Describe the intervention	FREETEXT
*Briefly describe the intervention, what participants are offered and any important factors such as conditionality, nature of housing, case management, substance abuse treatment included and so forth*.	
Theory of change	FREETEXT
*How does the intervention aim to bring about change? What is the underlying theoretical rationale for why the intervention might work to improve outcomes?*	
*If not specified write* “*not specified*”	
What is the size of accommodation/How many beds?	FREETEXT
Duration of treatment period from start to finish	FREETEXT
*In the dosage items, we are interested in the amount of treatment received by the participants. If the treatment was delivered directly to participants, the authors will probably provide at least some information about dosage and you can code these items accordingly. If minimal information is provided, you should try to give estimates for these items if you can come up with a reasonable estimate*.	
Timing	1. Once a month 2. Less than weekly 3. Once a week 4. 1–2 times a week 5. 2 imes a week 6. 2–3 times a week 7. 3 imes a week 8. 3‐4 times a week 9. Times a week 10. Daily contact
*Frequency of contact between participants and provider/program activity*	
	Cannot estimate
Length of each individual session	FREETEXT
*How long does each contact/session last?*	
Study Personnel	1. Graduate researcher 2. Grad/undergrad students 3. Author 4. Homelessness professional
*The primary individual/s who have direct contact with the participants served by the program*.	
	*Includes case manager, social worker, outreach worker*
*If the report is the author's dissertation (or based on the author's dissertation), then code as* “*Graduate Researcher*”.	
	5. Peers 6. Interventionist (not hired by researcher) 7. Interventionist (hired by researcher) 8. Self‐directed 9. Medical professionals 10. Other (please specify)
*If the delivery is performed by graduate or undergraduate students assisting the author then select* “*Grad/Undergrad Students*”.	
*Code “Self‐directed” for studies where electronic/computer programs are used*.	
*If the intervention is solely environmental i.e. community housing, then code “environmental change*”	
	Not Specified
Did provider receive specialised training?	1. Yes 2. The interventionist IS program developer 3. No
*This refers to whether or not the “interventionist” received specialised training to equip them to deliver the intervention proficiently*.	
	Not specified
Resource requirements	FREETEXT
*Time, staff, housing provision and so forth*	
Cost	FREETEXT

4a. Study design	
Design	4. Randomised control trial
*The studies included in all reviews must include an intervention group and at least one untrained control group. Control groups can include placebo, no treatment, waitlist or treatments vs “treatment as usual.” Any study which includes one group pretest/posttest or in which a treatment group is only compared to another treatment group will not be eligible for inclusion*.	*Individual or cluster randomised*
	4. Nonrandomised control trial
What do control subjects receive?	1. Placebo 2. Treatment as usual 3. No treatment
1. *Placebo (or attention) treatment. Group gets some attention or a sham treatment* 2. *Treatment as usual. Group gets “usual” handling instead of some special treatment*. 3. *No treatment. Group gets no treatment at all*.	
	Not specified
Unit of allocation	1. Individual 2. Group 3. Regions 4. Other (please specify)
*Individual (i.e., some were assigned to treatment group, some to comparison group)*	
*Group (i.e., whole subsets assigned to treatment and comparison groups)*	
	Not Specified
*Regions (i.e., region assigned as an intact unit)*	
Method of assignment	1. Randomly after matching 2. Randomly without matching 3. Regression discontinuity design 4. Cluster assigned 5. Wait list control 6. Nonrandom, but matched 7. Other (please specify)
*Method of group assignment. How participants/units were assigned to groups. This item focuses on the initial method of assignment to groups, regardless of subsequent degradations due to attrition, refusal, and so forth, prior to treatment onset*.	
1. *Randomly after matching, yoking, stratification, blocking, etc. The entire sample is matched or blocked first, then assigned to treatment and comparison groups within pairs or blocks. This does not refer to blocking after treatment for the data analysis*. 2. *Randomly without matching, etc. This also includes cases when every other person goes to the control group*. 3. *Regression discontinuity design: quantitative cutting point defines groups on some continuum (this is rare)*. 4. *Cluster assigned, this is to be used in cluster assignment studies only, specify the number of clusters in the treatment group and the number of clusters in control*. 5. *Wait list control or other quasirandom procedure presumed to produce comparable groups (no obvious differences). This applies to groups which have individuals apparently randomly assigned by some naturally occurring process, e.g., first person to walk in the door. The key here is that the procedure used to select groups does not involve individual characteristics of persons so that the groups generated should be essentially equivalent*. 6. *Nonrandom, but matched: Matching refers to the process by which comparison groups are generated by identifying individuals or groups that are comparable to the treatment group using various characteristics of the treatment group. Matching can be done individually, e.g., by selecting a control subject for each intervention subject who is the same age, gender, and so forth, or on a group basis*.	
	Not Specified
Was there >20% attrition in either/both groups?	FREETEXT
*Attrition occurs when participants are lost from an intervention over time or over a series of sequential processes. Studies may describe this as “lost to follow‐up,” or “drop outs*.”	

4b. Nonrandom studies	
How were groups matched?	1. Matched on pretest measure 2. Matched on personal characteristics 3. Matched on demographics 4. Groups were not matched 5. Other (please specify)
*If matching was used prior to assignment of condition, how were groups matched?*	
	Not specified
Was the equivalence of groups tested at pretest?	FREETEXT
Results of statistical comparisons of pretest differences	1. No statistically significant differences 2. Significant differences judged unimportant by coder 3. Significant differences judged of uncertain importance by coder 4. Significant differences judged important by coder 5. Other (please specify)
Were there pretest adjustments?	FREETEXT

5. Qualitative information	
Qualitative methods used	FREETEXT
Data analysis technique and procedure	FREETEXT
Was the intervention implemented as intended?	1. Yes 2. No
	Not specified
How was this measured?	FREETEXT
What implementation and process factors impact intervention delivery?	1. Contextual factors 2. Policy makers/funders 3. Programme managers/Implementing agency, 4. Staff/case workers 5. Recipients

6. Assessing quality in RCTs (Cochranes ROB2 tool)	
**Domain 1:** Risk of bias arising from the randomization process	
11. Was the allocation sequence random?	1. Yes 2. Probably yes 3. Probably No 4. No
12. Was the allocation sequence concealed until participants were enrolled and assigned to interventions?	1. Yes 2. Probably yes 3. Probably No 4. No
13. Did baseline differences between intervention groups suggest a problem with the randomization process?	1. Yes 2. Probably yes 3. Probably No 4. No
Risk‐of‐bias judgement	1. Low 2. High 3. Some concerns
Optional: What is the predicted direction of bias arising from the randomization process?	1. Favours experimental 2. Favours comparator 3. Towards null 4. Away from null 5. Unpredictable
**Domain 2:** Risk of bias due to deviations from the intended interventions (effect of assignment to intervention)	
21. Were participants aware of their assigned intervention during the trial?	1. Yes 2. Probably yes 3. Probably No 4. No
22. Were carers and people delivering the interventions aware of participants' assigned intervention during the trial?	1. Yes 2. Probably yes 3. Probably No 4. No
23. If Y/PY/NI to 2.1 or 2.2: Were there deviations from the intended intervention that arose because of the experimental context?	1. Yes 2. Probably yes 3. Probably No 4. No
24. If Y/PY to 2.3: Were these deviations from intended intervention balanced between groups?	1. Yes 2. Probably yes 3. Probably No 4. No
25. If N/PN/NI to 2.4: Were these deviations likely to have affected the outcome?	1. Yes 2. Probably yes 3. Probably No 4. No
26. Was an appropriate analysis used to estimate the effect of assignment to intervention?	1. Yes 2. Probably yes 3. Probably No 4. No
27. If N/PN/NI to 2.6: Was there potential for a substantial impact (on the result) of the failure to analyse participants in the group to which they were randomized?	1. Yes 2. Probably yes 3. Probably No 4. No
Risk‐of‐bias judgement	1. Low 2. High 3. Some concerns
Optional: What is the predicted direction of bias due to deviations from intended interventions?	1. Favours experimental 2. Favours comparator 3. Towards null 4. Away from null 5. Unpredictable
**Domain 3:** Missing outcome data	
31. Were data for this outcome available for all, or nearly all, participants randomized?	1. Yes 2. Probably yes 3. Probably No 4. No
32. If N/PN/NI to 3.1: Is there evidence that result was not biased by missing outcome data?	1. Yes 2. Probably yes 3. Probably No 4. No
33. If N/PN to 3.2: Could missingness in the outcome depend on its true value?	1. Yes 2. Probably yes 3. Probably No 4. No
34. If Y/PY/NI to 3.3: Do the proportions of missing outcome data differ between intervention groups?	1. Yes 2. Probably yes 3. Probably No 4. No
35. If Y/PY/NI to 3.3: Is it likely that missingness in the outcome depended on its true value?	1. Yes 2. Probably yes 3. Probably No 4. No
Risk‐of‐bias judgement	1. Low 2. High 3. Some concerns
Optional: What is the predicted direction of bias due to missing outcome data?	1. Favours experimental 2. Favours comparator 3. Towards null 4. Away from null 5. Unpredictable
**Domain 4:** Risk of bias in measurement of the outcome	
41. Was the method of measuring the outcome inappropriate?	1. Yes 2. Probably yes 3. Probably No 4. No
42. Could measurement or ascertainment of the outcome have differed between intervention groups?	1. Yes 2. Probably yes 3. Probably No 4. No
43. If N/PN/NI to 4.1 and 4.2: Were outcome assessors aware of the intervention received by study participants?	1. Yes 2. Probably yes 3. Probably No 4. No
44. If Y/PY/NI to 4.3: Could assessment of the outcome have been influenced by knowledge of intervention received?	1. Yes 2. Probably yes 3. Probably No 4. No
45. If Y/PY/NI to 4.4: Is it likely that assessment of the outcome was influenced by knowledge of intervention received?	1. Yes 2. Probably yes 3. Probably No 4. No
Risk‐of‐bias judgement	1. Low 2. High 3. Some concerns
Optional: What is the predicted direction of bias in measurement of the outcome?	1. Favours experimental 2. Favours comparator 3. Towards null 4. Away from null 5. Unpredictable
**Domain 5:** Risk of bias in selection of the reported result	
51. Was the trial analysed in accordance with a prespecified plan that was finalized before unblinded outcome data were available for analysis?	1. Yes 2. Probably yes 3. Probably No 4. No
Is the numerical result being assessed likely to have been selected, on the basis of the results, from…	
52. … multiple outcome measurements (e.g., scales, definitions, time points) within the outcome domain?	1. Yes 2. Probably yes 3. Probably No 4. No
53. … multiple analyses of the data?	1. Yes 2. Probably yes 3. Probably No 4. No
Risk‐of‐bias judgement	1. Low 2. High 3. Some concerns
Optional: What is the predicted direction of bias due to selection of the reported result?	1. Favours experimental 2. Favours comparator 3. Towards null 4. Away from null 5. Unpredictable
**Overall risk of bias**	
Risk‐of‐bias judgement	1. Low 2. High 3. Some concerns

7. Assessing quality in nonrandom control trials (ROBINS‐I tool)	
**Bias due to confounding**	
11. Is there potential for confounding of the effect of intervention in this study?	1. Yes 2. Probably yes 3. Probably No 4. No
**If N/PN to 1.1:** the study can be considered to be at low risk of bias due to confounding and no further signalling questions need be considered	
**If Y/PY to 1.1**: determine whether there is a need to assess time‐varying confounding:	1. Yes 2. Probably yes 3. Probably No 4. No
12. Was the analysis based on splitting participants' follow up time according to intervention received?	1. Yes 2. Probably yes 3. Probably No 4. No
**If N/PN**, answer questions relating to baseline confounding (1.4–1.6)	
**If Y/PY**, go to question 1.3.	
13. Were intervention discontinuations or switches likely to be related to factors that are prognostic for the outcome?	1. Yes 2. Probably yes 3. Probably No 4. No
**If N/PN**, answer questions relating to baseline confounding (1.4–1.6)	
**If Y/PY**, answer questions relating to both baseline and time‐varying confounding (1.7 and 1.8)	
**Questions relating to baseline confounding only**	
14. Did the authors use an appropriate analysis method that controlled for all the important confounding domains?	1. Yes 2. Probably yes 3. Probably No 4. No
15. **If Y/PY to 1.4**: Were confounding domains that were controlled for measured validly and reliably by the variables available in this study?	1. Yes 2. Probably yes 3. Probably No 4. No
16. Did the authors control for any postintervention variables that could have been affected by the intervention?	1. Yes 2. Probably yes 3. Probably No 4. No
**Questions relating to baseline and time‐varying confounding**	
17. Did the authors use an appropriate analysis method that controlled for all the important confounding domains and for time‐varying confounding?	1. Yes 2. Probably yes 3. Probably No 4. No
18. If **Y/PY** to 1.7: Were confounding domains that were controlled for measured validly and reliably by the variables available in this study?	1. Yes 2. Probably yes 3. Probably No 4. No
Risk‐of‐bias judgement	1. Low 2. Moderate 3. Serious 4. Critical
Optional: What is the predicted direction of bias due to confounding?	1. Favours experimental 2. Favours comparator 3. Unpredictable
**Bias in selection of participants into the study**	
21. Was selection of participants into the study (or into the analysis) based on participant characteristics observed after the start of intervention?	1. Yes 2. Probably yes 3. Probably No 4. No
**If N/PN to 2.1:** go to 2.4	
22. **If Y/PY to 2.1**: Were the postintervention variables that influenced selection likely to be associated with intervention?	1. Yes 2. Probably yes 3. Probably No 4. No
23. **If Y/PY to 2.2**: Were the postintervention variables that influenced selection likely to be influenced by the outcome or a cause of the outcome?	1. Yes 2. Probably yes 3. Probably No 4. No
24. Do start of follow‐up and start of intervention coincide for most participants?	1. Yes 2. Probably yes 3. Probably No 4. No
25. **If Y/PY to 2.2 and 2.3, or N/PN to 2.4**: Were adjustment techniques used that are likely to correct for the presence of selection biases?	1. Yes 2. Probably yes 3. Probably No 4. No
Risk‐of‐bias judgement	1. Low 2. Moderate 3. Serious 4. Critical
Optional: What is the predicted direction of bias due to selection of participants into the study?	1. Favours experimental 2. Favours comparator 3. Towards null 4. Away from null 5. Unpredictable
**Bias in classification of interventions**	
31. Were intervention groups clearly defined?	1. Yes 2. Probably yes 3. Probably No 4. No
32. Was the information used to define intervention groups recorded at the start of the intervention?	1. Yes 2. Probably yes 3. Probably No 4. No
33. Could classification of intervention status have been affected by knowledge of the outcome or risk of the outcome?	1. Yes 2. Probably yes 3. Probably No 4. No
Risk‐of‐bias judgement	1. Low 2. Moderate 3. Serious 4. Critical
Optional: What is the predicted direction of bias due to classification of interventions?	1. Favours experimental 2. Favours comparator 3. Towards null 4. Away from null 5. Unpredictable
**Bias due to deviations from intended interventions**	
If your aim for this study is to assess the effect of assignment to intervention, answer questions 4.1 and 4.2	
41. Were there deviations from the intended intervention beyond what would be expected in usual practice?	1. Yes 2. Probably yes 3. Probably No 4. No
42. **If Y/PY to 4.1**: Were these deviations from intended intervention unbalanced between groups *and* likely to have affected the outcome?	1. Yes 2. Probably yes 3. Probably No 4. No
If your aim for this study is to assess the effect of starting and adhering to intervention, answer questions 4.3–4.6	
43. Were important co‐interventions balanced across intervention groups?	1. Yes 2. Probably yes 3. Probably No 4. No
44. Was the intervention implemented successfully for most participants?	1. Yes 2. Probably yes 3. Probably No 4. No
45. Did study participants adhere to the assigned intervention regimen?	1. Yes 2. Probably yes 3. Probably No 4. No
46. **If N/PN to 4.3, 4.4 or 4.5**: Was an appropriate analysis used to estimate the effect of starting and adhering to the intervention?	1. Yes 2. Probably yes 3. Probably No 4. No
Risk‐of‐bias judgement	1. Low 2. Moderate 3. Serious 4. Critical
Optional: What is the predicted direction of bias due to deviations from the intended interventions?	1. Favours experimental 2. Favours comparator 3. Towards null 4. Away from null 5. Unpredictable
**Bias due to missing data**	
51. Were outcome data available for all, or nearly all, participants?	1. Yes 2. Probably yes 3. Probably No 4. No
52. Were participants excluded due to missing data on intervention status?	1. Yes 2. Probably yes 3. Probably No 4. No
53. Were participants excluded due to missing data on other variables needed for the analysis?	1. Yes 2. Probably yes 3. Probably No 4. No
54. **If PN/N to 5.1, or Y/PY to 5.2 or 5.3**: Are the proportion of participants and reasons for missing data similar across interventions?	1. Yes 2. Probably yes 3. Probably No 4. No
55. **If PN/N to 5.1, or Y/PY to 5.2 or 5.3**: Is there evidence that results were robust to the presence of missing data?	1. Yes 2. Probably yes 3. Probably No 4. No
Risk of bias judgement	1. Low 2. Moderate 3. Serious 4. Critical
Optional: What is the predicted direction of bias due to missing data?	1. Favours experimental 2. Favours comparator 3. Towards null 4. Away from null 5. Unpredictable
**Bias in measurement of outcomes**	
61. Could the outcome measure have been influenced by knowledge of the intervention received?	1. Yes 2. Probably yes 3. Probably No 4. No
62. Were outcome assessors aware of the intervention received by study participants?	1. Yes 2. Probably yes 3. Probably No 4. No
63. Were the methods of outcome assessment comparable across intervention groups?	1. Yes 2. Probably yes 3. Probably No 4. No
64. Were any systematic errors in measurement of the outcome related to intervention received?	1. Yes 2. Probably yes 3. Probably No 4. No
Risk of bias judgement	1. Low 2. Moderate 3. Serious 4. Critical
Optional: What is the predicted direction of bias due to measurement of outcomes?	1. Favours experimental 2. Favours comparator 3. Towards null 4. Away from null 5. Unpredictable
**Bias in selection of the reported result**	
Is the reported effect estimate likely to be selected, on the basis of the results, from…	
71. … multiple outcome measurements within the outcome domain?	1. Yes 2. Probably yes 3. Probably No 4. No
72. … multiple analyses of the intervention‐outcome relationship?	1. Yes 2. Probably yes 3. Probably No 4. No
73. … different subgroups?	1. Yes 2. Probably yes 3. Probably No 4. No
Risk of bias judgement	1. Low 2. Moderate 3. Serious 4. Critical
Optional: What is the predicted direction of bias due to selection of the reported result?	1. Favours experimental 2. Favours comparator 3. Towards null 4. Away from null 5. Unpredictable
**Overall risk of bias**	
Risk‐of‐bias judgement	1. Low 2. Moderate 3. Serious 4. Critical

8. Assessing quality in qualitative studies (White and Keenan tool)	
Are the evaluation questions clearly stated?	1. Yes 2. No
Is the qualitative methodology described?	1. Yes 2. No
Is the qualitative methodology appropriate to address the evaluation questions?	1. Yes 2. No 3. Insufficient detail
Is the recruitment or sampling strategy described?	1. Yes 2. No
Is the recruitment or sampling strategy appropriate to address the evaluation questions?	1. Yes 2. No 3. Insufficient detail
Are the researcher's own position, assumptions and possible biases outlined?	1. Yes 2. No
Have ethical considerations been sufficiently considered?	1. Yes 2. No 3. Insufficient detail
Is the data analysis approach adequately described?	1. Yes 2. No
Is the data analysis sufficiently rigorous?	1. Yes 2. No
Is there a clear statement of findings?	1. Yes 2. No
Are the research findings useful?	1. Yes 2. No
